# Combined Corneal Cross-Linking and Myoring Implantation in Advanced Keratoconus: Femtosecond Laser versus Manual Dissection

**DOI:** 10.1155/2021/6673842

**Published:** 2021-09-02

**Authors:** Ahmed Ibrahim Basiony, Moataz Fayez ElSawy, Mahmoud Mohamed Ismail, Mohamed Samy Abd ElAziz, Mahmoud Tawfik KhalafAllah, Adel Galal Zaky

**Affiliations:** ^1^Department of Ophthalmology, Faculty of Medicine, Menoufia University, Menoufia, Egypt; ^2^Department of Ophthalmology, Faculty of Medicine, Al-Azhar University, Cairo, Egypt

## Abstract

**Background:**

Intrastromal corneal ring segments are widely adopted for keratoconus management. However, the complete ring (Myoring) was proposed to be superior in advanced cases. Myoring can be implanted either via femtoassisted or manual dissection techniques. A comparison between both techniques can delineate any differences in the outcomes.

**Methods:**

This was a prospective interventional case series study. Sixty-four eyes with progressive advanced keratoconus were enrolled: 36 and 28 had femtoassisted or manual Myoring, respectively. Uncorrected visual acuity (UCVA), corrected distance visual acuity (CDVA), maximal keratometry (*K*_max_), spherical equivalent (SE) and corneal thinnest location were measured in all eyes preoperatively and at one, six, and 12 months postoperatively. Epi-off corneal cross-linking (CXL) was performed eight weeks after Myoring implantation for all cases.

**Results:**

Femtoassisted Myoring dissection significantly improved UCVA and CDVA from 0.1 ± 0.06 and 0.18 ± 0.1 preoperatively to 0.29 ± 0.08 and 0.43 ± 0.1 at 12 months. Also, manual technique similarly enhanced UCVA and CDVA from 0.11 ± 0.05 and 0.2 ± 0.1 preoperatively to 0.27 ± 0.2 and 0.4 ± 0.2 at 12 months. In terms of safety, while no cases of ring extrusion were encountered with the femtoassisted technique, six (21.4%) cases of extrusion were encountered in the manual group.

**Conclusion:**

Femtoassisted or manual Myoring technique followed by CXL is an effective choice for advanced progressive keratoconus. Although it did not reach a statistical significance, the high extrusion rate with manual dissection is a red flag to be considered.

## 1. Background

Keratoconus (KC) is an ectatic corneal disorder characterized by progressive corneal thinning with subsequent protrusion, irregular astigmatism, and diminished vision [1]. Most patients with mild forms of the disease can be managed with spectacles or contact lenses. However, when these measures fail to provide adequate vision or patients can no longer tolerate contact lenses, penetrating keratoplasty (PKP) is a surgical alternative with high success rates, but also with potential complications [2, 3].

Intrastromal corneal ring segments (ICRS) have been widely adopted as an additive surgical procedure for KC correction [4] through flattening the central cornea via an “arc-shortening” effect on the corneal lamellae [5]. The concept of a full corneal intrastromal ring as an additive refractive technique for the correction of myopia was first proposed by Reynolds in 1978 [6]. Extensively enhanced by Daxer [7], implantation of a full corneal intrastromal implantation system (CISIS) has been considered for KC management [8–10].

For CISIS, tunnel creation can be carried out either via a femtosecond laser-assisted or manual dissection technique. The low cost of manual dissection is appealing for settings where advanced technology is lacking. However, since first applied [11], the femtosecond laser showed superiority and could avoid the potential inaccuracies of the manual technique; hence making the procedure safer, reliable, and more accurate [12].

In this study, we aimed to evaluate the effectiveness and safety of the Myoring (Dioptex, GmbH, Linz, Austria) followed by corneal cross-linking (CXL) in advanced KC. In addition, we aimed to compare the femtoassisted and manual dissection techniques in terms of visual, refractive, tomographic, and safety outcomes.

## 2. Patients and Methods

This was a prospective case series study conducted at a private center in Cairo, Egypt, and Menoufia University Hospitals, Egypt, from September 2016 to September 2018. All study procedures were approved by the Ethical Committee of Menoufia Faculty of Medicine and were in accordance with the Declaration of Helsinki. A well-informed consent was obtained from all enrolled participants. We included patients with progressive advanced keratoconus diagnosed with Scheimpflug imaging (WaveLight® Allegretto Oculyzer). Progressive KC was defined as increase in maximum keratometry (*K*_max_) of 1.00 diopter (*D*) or loss of 1 line of corrected distance visual acuity (CDVA) over 6 months. Advanced KC was defined as stage III on Amsler-Krumeich classification. Allocation of patients to either technique was solely based on the corneal thinnest location: not less than 400 micrometers (*μ*m) for manual dissection and not less than 380 *μ*m for femtosecond-assisted implantation. For those with thinnest location ≥400 *μ*m, when either technique fits, a quasirandomized method was applied.

### 2.1. Preoperative Evaluation

For all patients, uncorrected visual acuity (UCVA), CDVA, intraocular pressure, fundus examination, and corneal tomography (WaveLight® Allegretto Oculyzer, GmbH, Erlangen, Germany) were carried out. While no consensus exists for appropriate diameter selection, we followed the nomogram described by Jadidi et al. which takes into account the spherical equivalent (SE) and the keratometric readings [8]. For appropriate thickness selection, we implanted 300 and 320 *μ*m Myoring for SE below or above - 6 D, respectively.

### 2.2. Surgical Technique

Myoring (Dioptex, GmbH, Linz, Austria) implantation was carried out either assisted by a femtosecond laser or with manual dissection. Corneal cross-linking (CXL) was performed for all patients eight weeks after uneventful Myoring implantation.

#### 2.2.1. Femtoassisted Technique

Femtosecond Laser (Victus SW, Technolas Perfect Vision, GmbH, Munich, Germany) was used for the creation of an almost entirely closed intrastromal pocket of 9 mm in diameter and 300 *μ*m in depth. The pocket space was gently formed by passing a spatula through the upper incision between the closed flap and the bed. The Myoring was inserted using special forceps with a groove to accommodate the ring and then adjusted to be centered on the corneal reflex. The built-in anterior segment optical coherence tomography (AS-OCT) was used to adjust the ring position intraoperatively.

#### 2.2.2. Manual Dissection

The exact ring pocket was labelled with a marker and the entry incision was made at 12 o'clock with a diamond knife. Guided by intraoperative pachymetry, pocket depth was set to be at 80% of the corneal thickness at the incision site, and then the incision was widened. After that, intrastromal manual dissection was performed using a 1.25 mm crescent blade with guarded advancement to involve the whole marked area. The Myoring was inserted using special forceps with a groove to accommodate the ring and then adjusted to be centered on the corneal reflex and 1 mm away from the incision site.

### 2.3. Corneal Cross-Linking (CXL)

Accelerated epi-off CXL (10 minutes program) was performed for all cases 8 weeks after Myoring implantation. Starting with Benoxinate instillation for topical anesthesia, corneal epithelium was manually removed after applying alcohol 20% for 20 second in surgical well over the cornea. Riboflavin 0.1% in 20% dextran solution (Ricrolin; Sooft, Montegiorgio, Italy) was instilled every 2 minutes for 30 minutes, and then every 2 minutes during UVA exposure. The cornea was exposed to UVA 370 nm light (UV-X System; Peschke Meditrade GmbH, Hünenberg, Switzerland) for 10 minutes at an irradiance of 9 mW/cm2. After that, a therapeutic contact lens was applied. Topical antibiotic and steroid (Fortymox® q.i.d and Predforte® q.i.d) were prescribed for all patients.

### 2.4. Main Outcome Measures

UCVA, CDVA, SE, *K*_max_, and corneal thinnest location thickness were assessed at 1, 6, and 12 months after Myoring in all patients.

### 2.5. Sample Size Calculation

Sample size was calculated using Power Analysis Sample Size software (version 15, NCSS, LLC) setting the type 1 error (a) at 0.05 (95% confidence interval) and the power [1−b] at 0.8. Sample size was calculated to detect a difference of at least 0.1 decimal difference in CDVA between both groups. This was agreed upon by the authors as a meaningful clinical difference and hence set as the primary outcome. Getting the anticipated means for either technique from previous literature [8, 11, 13, 14], the sample size calculation was set to be at least 25 eyes per group.

### 2.6. Statistical Analysis

Data analysis was conducted using SPSS v.24 software (SPSS Inc., Chicago, IL, USA). Continuous variables were presented as mean ± standard deviation. Normal distribution of the data was checked by the Kolmogorov-Smirnov test. Student's *t*-test was used to compare the means of the outcomes between both groups. The paired *t*-test was used to compare the preoperative and postoperative values. Chi-square test was used to compare categorical variables. *P* value < 0.05 was considered statistically significant in all these tests.

## 3. Results

In the current study, 64 eyes have undergone Myoring implantation. The mean age was 27.44 ± 5.4 years. Myoring implantation was carried out in 36 eyes using a femtosecond laser-assisted technique and by manual implantation in 28 eyes.

### 3.1. Visual and Refractive Outcomes

The mean preoperative UCVA and CDVA were 0.1 ± 0.06 and 0.15 ± 0.1, respectively, and improved after 1 month postoperatively to 0.2 ± 0.06 and 0.3 ± 0.11, respectively (*P*=0.001), 0.23 ± 0.06 and 0.41 ± 0.10, respectively, after 6 months (*P*=0.001), and after 12 months to 0.26 ± 0.08 and 0.4 ± 0.10, respectively (*P*=0.001). Regarding the SE, the preoperative mean SE was −7.3 ± 3.32, which decreased to −2.41 ± 3.91 after one month (*P*=0.001), −2.3 ± 3.85 after 6 months (*P*=0.001), and to −1.7 ± 3.32 after 12 months (*P*=0.001).

### 3.2. Tomographic Outcomes

Preoperative mean Kmax was 56.92 ± 3.39 *D*, which changed to 51.70 ± 3.44 *D* after 1 month postoperatively (*P*=0.001), to 50.47 ± 3.26 after 6 months postoperatively (*P*=0.001), and to 50.40 ± 3.12 *D* after 12 months (*P*=0.001). Mean preoperative corneal thinnest location was 439.11 ± 32.47 *μ*m which increased to 453.00 ± 37.40 *μ*m after 1 month postoperatively (*P*=0.001) and to 450.22 ± 35.48 *μ*m after 6 months postoperatively (*P*=0.001) and became 454.72 ± 34.1 *μ*m after 12 months postoperatively (*P*=0.001).

### 3.3. Femtoassisted versus Manual Dissection Technique

To ensure the reliability of the comparison, baseline demographic features were compared between both groups. The mean age was 27.6 and 23.7 years in the femtoassisted and manual groups respectively (*P*=0.07). Gender distribution was also comparable between both groups (*P*=0.7). All enrolled participants were classified as advanced keratoconus with a mean Kmax of 58.7 and 57 *D* in the femtoassisted and manual groups, respectively (*P*=0.9). As well, no significant differences were detected for visual and refractive baseline features between both groups as shown in Table 1.

Both techniques could significantly enhance the visual, refractive, and tomographic outcomes. We will highlight the final 12-month outcomes for both groups. In terms of vision, UCVA has improved to 0.29 and 0.27 decimal units in the femtoassisted and the manual groups respectively (*P*=0.6). Similarly, CDVA has improved to 0.43 and 0.4 in the femtoassisted and manual groups respectively (*P*=0.09).

**Figure 1:**
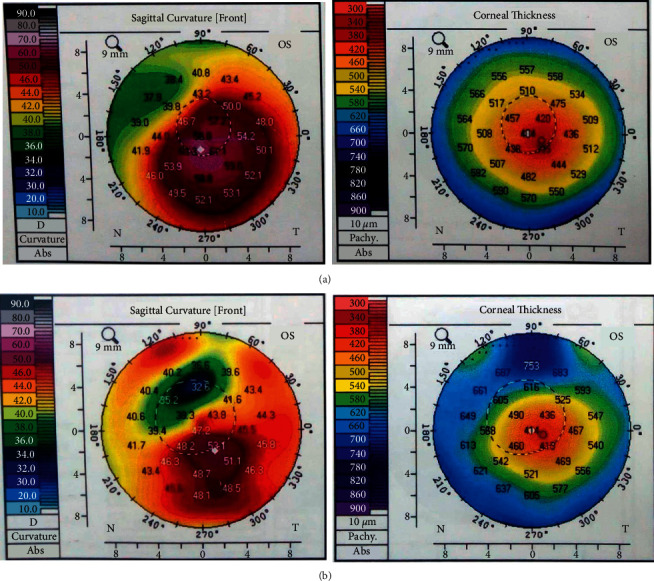
Corneal tomography before (a) and after (b) femtoassisted Myoring implantation.

**Figure 2:**
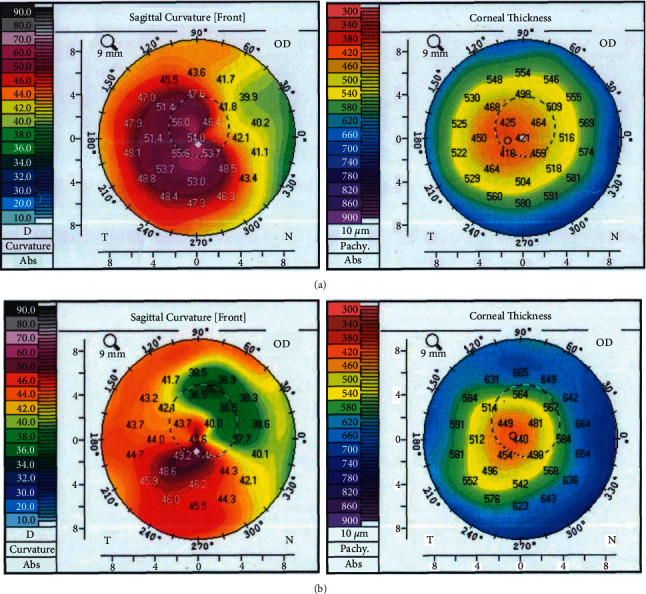
Corneal tomography before (a) and after (b) manual Myoring implantation.

Refractive and tomographic outcomes were also comparable between both groups. At 12 months, SE has been reduced to −1.06 and −2.00 in the femtoassisted and manual groups respectively (*P*=0.6). *K*max was not different from the prior outcomes (51.1 *D* versus 49.7 *D*) in the femtoassisted and manual groups respectively (*P*=0.3). Thinnest location thickness has increased in the femtoassisted and manual groups to 455 and 454 *μ*m respectively (*P*=0.4).  Table 2 plots the postoperative outcomes for both groups. Figures 1 and 2 show the pre- and postoperative tomography for both groups.

### 3.4. Safety Profile

In the current study, no intraoperative complications were encountered in either group. Cumulative incidence of postoperative adverse events was 6 (16.6%) and 8 (28.5%) in the femtoassisted and manual groups, respectively (*P*=0.4).  Table 3 shows the different adverse events and their incidence for both techniques. Regarding the CXL, no intra-or postoperative complications were detected.

## 4. Discussion

As an additive procedure, corneal rings have been proposed for KC correction [4], aiming to eliminate or at least delay the need for keratoplasty [15]. Implantation of a full corneal intrastromal ring (Myoring) has been described by Daxer for use in the correction of KC [16]. Nonetheless, the paucity of reports on Myoring raised the need for further evaluations. According to the topology concept, if a soft surface gets tightened to a closed rigid structure, any point on such surface is determined by the circumferential shape of that structure. Therefore, Myoring is perfectly suited to regularize the cornea in its optical center regardless the type of KC or the irregularity pattern prior to its implantation [8, 13, 14].

In the present study, Myoring implantation showed significant improvement of both UCVA and CDVA from 0.07 ± 0.01 and 0.15 ± 0.11 to 0.19 ± 0.1 and 0.31 ± 0.1, respectively (*P*=0.001) after 1 year. In addition, SE has significantly improved after 1-year follow-up period from −12.29 ± 3.32 to −4.0 ± 3.3 (*P*=0.001). This comes in agreement with the findings of Daxer et al., who reported statistically significant improvement in both UCVA and CDVA with Myoring [17]. Furthermore, Daxer et al. reported that the improved visual outcomes have been maintained for a long-term follow-up. The latter finding not only indicates a significant degree of visual rehabilitation, but also indicates a stabilization of the diseased cornea following Myoring implantation [17].

In our study, Myoring significantly attained corneal flattening evidenced by significant reduction in keratometric readings (*K* max) from 56.9 ± 3.4 *D* to 50.4 ± 3.1 *D* after 1 year (*P*=0.001). The increment in corneal thinnest location thickness in the present study can be attributed, at least in part, to the stromal collagen redistribution. This is in agreement with Jabbarvand et al. who reported central corneal flattening after Myoring implantation with a mean change of 6.9 *D* and significant increase in central corneal thickness [18].

In one of the early reports on long-term outcomes of manual Myoring implantation, Shwartz et al. followed up 42 eyes for ten years. Thirty-five eyes (83%) and 27 eyes (64%) could achieve a CDVA of at least 20/40 and 20/20, respectively. Further, 28 eyes (68%) were within 1 *D* of target refraction after 5 years. While this may be out of range compared to our series and other studies, the inclusion criteria by Shwartz et al. may have an explanation for this since they only included patients with at least 20/20 CDVA, spherical refractive error less than −4.5 *D*, and astigmatism less than −1.00 D. This is considerably different from our inclusion criteria. The mean value of visual gain or myopic reduction was not reported, which may confound the interpretation [19].

To extend the interpretation of our results, we highlight the three-year outcomes of manual technique for Myoring implantation combined with CXL reported by Bikbova and his team. Using microkeratome pocket maker, Myoring significantly enhanced UCVA and CDVA from 1.06 to 0.49 logarithm of minimum angle of resolution (LogMAR) to 0.3 and 0.28 after 36 months respectively. SE, as well, was significantly decreased from −7.8 to −1.6 D and the average *k* readings similarly were reduced from 50.2 to 40.8 *D* [20].

Given the high cost of a PocketMaker, it was not available for the present study. Instead, we used a diamond knife and a crescent blade to create the pocket which is significantly lower in cost. Our results are comparable to those reported by Pirhadi and his colleagues who compared the PocketMaker microkeratome versus Melles hook technique. Mean changes in preoperative and postoperative UCVA (LogMAR) for the PocketMaker and Melles hook groups were recorded at 0.75 ± 0.32 and 0.78 ± 0.33, respectively. Similarly, CDVA (LogMAR) mean changes were 0.27 ± 0.22 and 0.23 ± 0.22 in the PocketMaker and Melles hook, respectively, which are comparable to our series. Mean keratometry reduction was 6.1 ± 0.4 and 6.2 ± 3.55 in the PocketMaker and Melles hook, respectively. UCVA change (*P* = 0.76), CDVA change (*P* = 0.77), and K-means change (*P* = 0.69) showed that there was no statistically significant difference between both groups for any parameter [10].

The comparison between both techniques for Myoring implantation is scarce in the literature. Among those who compared the manual versus the femtoassisted technique, Daxer reported comparable outcomes for both techniques. Nonetheless, the small sample size (seven eyes per group) was a major limitation in that study [14]. For ring segments, Kubaloglu et al. found no statistically significant differences either in the visual or the refractive parameters between the manual and the femtosecond techniques [21]. On the opposite side, Piñero et al. showed that the results of the femtoassisted technique were better than the manual one [22].

Safety is another major determinant for deciding which technique to adopt. The high extrusion rate (21.4% in the manual versus 0% with femtoassisted) may be explained by the less precise depth evaluation in the manual group. In addition, only advanced KC cases were included, which impose more challenges for the manual dissection. Although it did not reach statistical significance, we consider the high extrusion rate as a red flag for consideration with the manual dissection.

Corneal CXL is widely applied with either complete or incomplete corneal rings for KC [23, 24]. The optimal sequence of and interval between ring implantation and CXL is controversial. In theory, stabilizing a flattened cornea may be more effective in terms of improving visual acuity. Studies with same-day, 3, or 7 months intervals between the ICRS and CXL achieved comparable clinical outcomes [25–28]. However, this issue was not investigated for Myoring implantation. So, in the present study, Myoring implantation was carried out first followed by CXL. A synergistic effect for CXL when combined with Myoring was suggested in previous studies [29, 30]. Bijbova reported significantly reduced SE and mean K in the Myoring with CXL Myoring + CXL compared to Myoring alone [20]. A similar stronger flattening effect was attained on the horizontal meridian when Myoring was combined with CXL [31]. Prospective studies with well-organized, homogenous protocols are required to establish a consensus for the optimal sequence and interval between the Myoring and CXL.

This study has some limitations to be in mind. First, assessment of aberrations and the quality of vision was not included. Various studies reported a high incidence of halos and glare after Myoring implantation. Out of 47 eyes with femtoassisted Myoring implantation, Mohebbi and his colleagues reported night glare and night halos in 51.1% and 55.3% of the included eyes, respectively [32]. For the manual technique, no reports could be found reporting the aberrations after Myoring implantation. Nonetheless, Pinero compared both techniques in ring segments, reporting a significant increase in high-order aberrations in the manual group in contrast to the femtoassisted one [22]. Such reports raise the need for a well-structured comparison between both techniques regarding aberrometric changes and the related quality of vision. Moreover, the lack of either intra-or postoperative AS-OCT was a hurdle for accurate depth evaluation in the manual dissection group. One more limitation was that the outcomes of Myoring alone were not evaluated, so that the additional effect of CXL cannot be precisely determined.

## Figures and Tables

**Table 1:** Baseline characteristics of study population.

Variable	Femtoassisted (*n* = 36)	Manual (*n* = 28)	*P* value
Age	27.6 ± 3.8	23.7 ± 3.5	0.07
UCVA	0.1 ± 0.06	0.11 ± 0.05	0.2
CDVA	0.18 ± 0.1	0.2 ± 0.1	0.4
MRSE (D)	−7.5 ± 4.5	−7.7 ± 4.4	0.1
*K*_max_ (D)	58.7 ± 3.8	57 ± 3.4	0.9
ThL (*μ*m)	438.8 ± 33.8	441.8 ± 29.7	0.2

D, diopters; ThL, thinnest corneal location; *μ*m, micrometer.

**Table 2:** Postoperative outcomes of both groups at follow-up visits.

Variable	1 month			6 months			12 months		
	Femtoassisted	Manual	*P*	Femtoassisted	Manual	*P*	Femtoassisted	Manual	*P*
UCVA	0.22 ± 0.12	0.21 ± 0.1	0.8	0.28 ± 0.13	0.24 ± 0.12	0.2	0.29 ± 0.08	0.27 ± 0.2	0.6
CDVA	0.36 ± 0.1	0.34 ± 0.1	0.6	0.42 ± 0.1	0.39 ± 0.2	0.8	0.43 ± 0.1	0.4 ± 0.2	0.9
MRSE (D)	−2.9 ± 1.8	−2.4 ± 1.5	0.5	−2.5 ± 1.7	−2.2 ± 1.2	0.7	−1.06 ± 1.6	−2.00 ± 1.1	0.6
*K*_max_ (D)	52.1 ± 2.4	50.6 ± 3.2	0.7	51.2 ± 3.3	49.9 ± 2.3	0.6	51.1 ± 3.7	49.7 ± 2.7	0.3
ThL (*μ*m)	459 ± 49.1	452 ± 35.8	0.6	455 ± 21.2	454 ± 36.7	0.5	455 ± 15.2	454 ± 39.9	0.4

D, diopters; ThL, thinnest corneal location; *μ*m, micrometer.

**Table 3:** Adverse events of both groups.

Type of complication		Femtoassisted (*N* = 36)	Manual (*N* = 28)	*P* value
Intraoperative		0 (0%)	0 (0%)	0.4
Postoperative	(i) Extrusion	0 (0%)	6 (21.4%)	
	(ii) Epithelial defect	0 (0%)	2 (7.1%)	
	(iii) White deposits	2 (5.6%)	0 (0%)	
	(iv) Infectious keratitis	2 (5.6%)	0 (0%)	
	(v) Vascularization	0 (0%)	0 (0%)	
	(vi) One-line decrease in DCVA	2 (5.6%)	0 (0%)	

## Data Availability

All the data generated or analyzed during this study are included in this published article.

## Abbreviations

AS-OCT: Anterior segment optical coherence tomography
CDVA: Corrected distance visual acuity
CISIS: Corneal intrastromal implantation system
CXL: Cross-linking
D: Diopter
ICRS: Intrastromal corneal ring segments
KC: Keratoconus
*K*_max_: Maximum keratometric reading
LogMAR: Logarithm of minimum angle of resolution
*μ*m: Micrometer
PKP: Penetrating keratoplasty
SE: Spherical equivalent
UCVA: Uncorrected visual acuity.
